# Mitochondrial Metabolomics of Sym1-Depleted Yeast Cells Revealed Them to Be Lysine Auxotroph

**DOI:** 10.3390/cells12050692

**Published:** 2023-02-22

**Authors:** Simon Lagies, Daqiang Pan, Daniel A. Mohl, Dietmar A. Plattner, Ian E. Gentle, Bernd Kammerer

**Affiliations:** 1 Core Competence Metabolomics, Hilde-Mangold-Haus, University of Freiburg, 79104 Freiburg, Germany; 2 Institute of Organic Chemistry, University of Freiburg, 79104 Freiburg, Germany; 3 Institute of Pharmaceutical Science, University of Freiburg, 79104 Freiburg, Germany; 4 Institute of Medical Microbiology and Hygiene, Medical Center–University of Freiburg, Faculty of Medicine, University of Freiburg, 79104 Freiburg, Germany; 5 BIOSS Centre for Biological Signalling Studies, University of Freiburg, 79104 Freiburg, Germany; 6 Spemann Graduate School of Biology and Medicine (SGBM), University of Freiburg, 79104 Freiburg, Germany

**Keywords:** compartment-specific metabolomics, mitochondria, yeast, sym1, lysine auxotroph, mitochondrial DNA depletion syndrome, MDDS

## Abstract

Metabolomics has expanded from cellular to subcellular level to elucidate subcellular compartmentalization. By applying isolated mitochondria to metabolome analysis, the hallmark of mitochondrial metabolites has been unraveled, showing compartment-specific distribution and regulation of metabolites. This method was employed in this work to study a mitochondrial inner membrane protein Sym1, whose human ortholog MPV17 is related to mitochondria DNA depletion syndrome. Gas chromatography–mass spectrometry-based metabolic profiling was combined with targeted liquid chromatography–mass spectrometry analysis to cover more metabolites. Furthermore, we applied a workflow employing ultra-high performance liquid chromatography–quadrupole time of flight mass spectrometry with a powerful chemometrics platform, focusing on only significantly changed metabolites. This workflow highly reduced the complexity of acquired data without losing metabolites of interest. Consequently, forty-one novel metabolites were identified in addition to the combined method, of which two metabolites, 4-guanidinobutanal and 4-guanidinobutanoate, were identified for the first time in *Saccharomyces cerevisiae*. With compartment-specific metabolomics, we identified *sym1*Δ cells as lysine auxotroph. The highly reduced carbamoyl-aspartate and orotic acid indicate a potential role of the mitochondrial inner membrane protein Sym1 in pyrimidine metabolism.

## 1. Introduction

Subcellular compartmentalization allows the spatial distribution of metabolites among different cell organelles so that individual metabolites can be involved in multiple biological processes simultaneously. Although compartment-specific metabolite distribution was shown over four decades ago [1], it has been for a long time a challenge to get further insights into the activity of subcellular metabolites. To elucidate subcellular compartmentalization, isolated mitochondria were investigated using metabolomic analyses [2,3,4,5,6], highlighting the extension of metabolomics from cellular to subcellular level. Consequently, the barrier of compartmentalization could be overcome by showing compartment-specific metabolite distribution and regulation upon genetic or environmental change [2,3,6].

Yeast Saccharomyces cerevisiae SYM1 is an ortholog of human MPV17, whose mutations cause mitochondrial DNA depletion syndrome (MDDS) [7,8]. Ninety-eight pathogenic variants of MPV17 have been reported till the end of 2017, presenting mostly with hepatic and neurologic manifestations [9]. However, the function of the protein MPV17 is still undefined. Several biological models, including mice, yeast, and zebrafish, have been applied to modulate the function of MPV17 [10,11,12,13,14], showing various abnormal phenotypes. While MPV17-deficient humans developed liver disease [9], Mpv17-deficient mice suffered from renal failure [15]. Mpv17-depleted mice showed deoxynucleotide insufficiency in liver mitochondria [16]. Furthermore, impaired folate-mediated one-carbon metabolism was observed in mitochondria of HeLa cells with reduced MPV17 expression [17], presenting depleted mitochondrial dTMP pools. Therefore, MPV17 was predicted to be involved in the transport of dTMP from the cytosol to the mitochondria [17]. This is similar to a yeast model investigated before, proposing the yeast ortholog Sym1 as a transporter for TCA cycle intermediates [11]. Moreover, Sym1 is reported to be a channel-forming protein that maintains structural and functional stability as well as membrane potential [11,12]. Recently, Sym1 was presented to be part of a high molecular weight complex with undefined function [18], leaving more questions to be explained.

Liquid chromatography (LC)–quadrupole time-of-flight (QTOF) mass spectrometry-based metabolomics has been widely applied in plant phytochemical analysis [19], nutrition science [20], and cancer biomarker discovery [21,22]. Although QTOF can supply accurate masses of analytes, it is still a challenge to identify metabolites. Furthermore, the ability to detect thousands of mass features highly increases the data complexity [23]. Importantly, LC–QTOF analysis complements well with untargeted GC–MS profiling, since its analytes do not have to be volatile and it does not have strict molecular weight restrictions. On the other hand, more compounds can be ionized by electron ionization and the following fragmentation reactions yield a high amount of fragments. Numerous fragment ions in combination with system independent retention indices obtained by GC make the identification process more robust.

Therefore, we applied mitochondrial metabolomics in this work to study the mitochondrial inner membrane protein Sym1 with an unknown function using GC–MS profiling, targeted LC–MS, and a U(H)PLC-QTOF/MS-based workflow focusing on only significantly changed metabolites. Consequently, fifty-one metabolites were identified as significantly changed, of which forty-one were novel compared to the combined method. With the help of a powerful chemometrics platform, this workflow highlights a strategy in identifying altered metabolites within a highly complex data matrix.

## 2. Materials and Methods

### 2.1. Yeast Culture and Mitochondria Isolation

*Saccharomyces cerevisiae* strains *sym1*Δ and the corresponding wildtype (WT) BY4741 were obtained from Euroscarf. The detailed cell culture condition and the isolation of mitochondria have been described before [2,3].

### 2.2. Phenotype Test of Sym1Δ Cells

Cultures of *sym1*Δ and WT were grown in liquid synthetic galactose medium (SC medium) (0.67% [*w*/*v*] yeast nitrogen base without amino acids (Becton, Dickinson and Company, Sparks, MD, USA), 0.77 g/L SC amino acids (MP Biomedicals, Irvine, CA, USA), 2% [*w*/*v*] galactose (Sigma-Aldrich, Taufkirchen, Germany)) overnight at 23 °C to exponential phase before 1 OD_600_ unit of each culture was taken. The cells were pelleted at room temperature at 4000 rpm for 5 min. After removal of the culture medium, the cells were washed and resuspended in 1 mL sterile dH_2_O, with which a tenfold serial dilution including five concentrations was made. A quantity of 3 µL of each concentration was plated on agar plates (SC medium + 3% agar (Sigma-Aldrich)) with or without a specific metabolite.

### 2.3. Combined GC–MS Profiling and Targeted LC–MS

Details of the combination of GC–MS profiling and the targeted analysis of amino acids and pyrimidine metabolism intermediates are provided in our previous work [3]. The raw data of GC–MS and the combination with LC–MS are displayed in Supplementary Table S1. 

MetaboAnalyst 5.0 was used for statistical analyses, including auto-scaling, principal component analysis, ANOVA followed by false-discovery rate correction, as well as heat map generation [24].

### 2.4. U(H)PLC–QTOF Workflow

**Figure 1:**
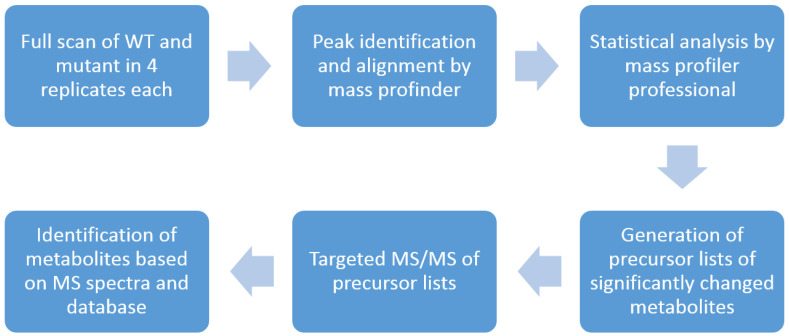
Overview of the U(H)PLC–QTOF based workflow. This workflow aims to identify significantly changed metabolites between two groups.

As shown in Figure 1, this workflow starts with a full-scan of WT and mutants using a U(H)PLC–QTOF instrument (6545, Agilent Technologies, Waldbronn, Germany). After data processing with Mass Profinder (Agilent Technologies) and Mass Profiler Professional (Agilent Technologies), precursor lists were generated including significantly changed metabolites. These precursor lists were applied to targeted MS/MS analyses to obtain the spectral information of the interested metabolites. Metabolites were identified by searching the spectra in the supplied library (Agilent Technologies) as well as from the literature.

Briefly, the dried sample pellets were resuspended in 100 µL of water before 5 µL of each was injected onto a HILIC-Z column (100 × 2.1 mm, Agilent Technologies) with the flow rate set to 0.6 mL/min. Buffer A was 10 mM ammonium formate in water while buffer B was 10 mM ammonium formate in 10/90 water/acetonitrile (LC–MS grade, Carl Roth, Karlsruhe, Germany). The following gradient was used: 100% B to 75% B in 10 min, 75% B to 60% B till 12 min, 60% B till 15 min, 60% B to 100% B till 17 min, post run 5 min. The mass spectrometer was operated in full-scan mode. All samples were analyzed in technical four replicates so that statistical analysis could be made. The data sets of WT and mutants were processed using Mass Profinder for peak picking and alignment with a 10-ppm mass accuracy and 0.25-retention time window before statistical analysis was done by Mass Profiler Professional. The significantly changed features were exported as precursor lists including accurate mass and retention time for further targeted MS/MS analyses. All chromatographic settings remained the same except that the mass spectrometer was operated in targeted MS/MS mode with the exported precursor lists. After the analysis with the inclusion lists for each sample, the data files were processed with MassHunter Qualitative Analysis (Agilent Technologies) by using Compound Discovery with Find by Targeted MS/MS. The acquired mass spectrum was searched in the supplied METLIN mass spectral library and the literature.

## 3. Results

### 3.1. GC–MS-Based Metabolic Profiling Combined with Targeted LC–MS

**Figure 2:**
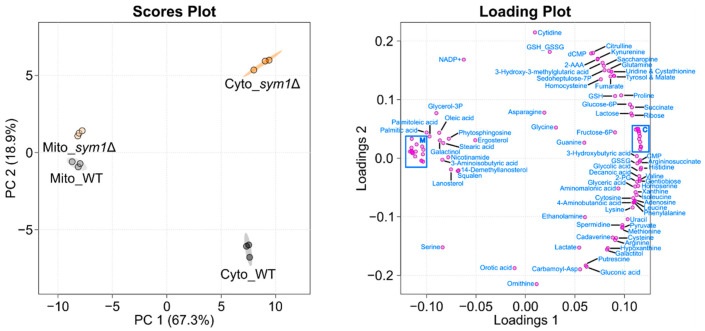
Principal Component analysis (PCA) score plot (**left**) and its loading plot (**right**). PC1 discriminates mitochondria (Mito) from the cytoplasm (Cyto), while PC2 discriminates *sym1*Δ from WT. The PCA loading plot shows how the individual metabolites contributes to the discrimination of groups. For readability, closely spaced metabolites only discriminating component 1 were not labeled but blue-rimmed. These were present in the C-cluster: 3-phosphoglyceric acid, alanine, AMP, aspartate, citrate, glutamate, Mannose-6P, phosphoenolpyruvate, threonine, trehalose, tryptophan, tyrosine, and UMP; and in the M-cluster: 1-monopalmitin, 1-octadecanol, 5-methyluridine, adenine, dCytidine, eicosanoic acid, ergosterol, glucose, hexacosanoic acid, lauric acid, maltose, methylphosphate, myo-inositol, myristic acid, NAD^+^, O-phosphocolamine, and pyroglutamate. N = 3.

**Figure 3:**
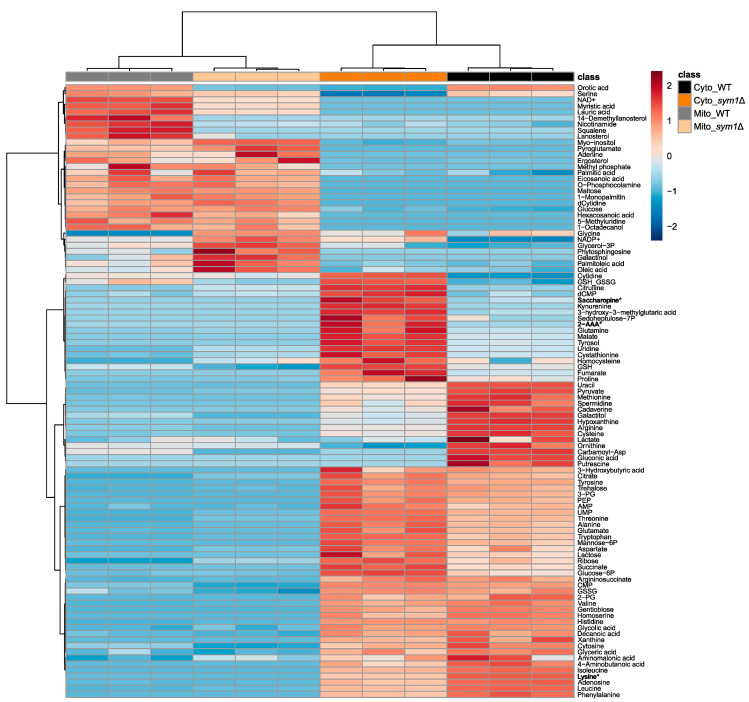
Heat map of significantly altered metabolites in mitochondria (Mito) and cytoplasm (Cyto), isolated from BY4741 (WT) and *sym1*Δ cells. Lysine biosynthesis intermediates, 2-aminoadipate and saccharopine, and lysine are highlighted in bold style with asterisk. Auto-scaled z-scores are displayed. Samples and metabolites were clustered with Ward’s method and Euclidean distance measure. N = 3.

To evaluate if the deletion of SYM1 causes compartment-specific metabolic alterations, the isolated mitochondria and cytoplasm were subjected to compartment-specific metabolomic analysis using combined GC–MS profiling and targeted LC–MS analysis. The annotated metabolites with their normalized intensities are displayed in Supplementary Table S1. As expected, the cytoplasm and mitochondria of *sym1*Δ cells showed different metabolic patterns. By analyzing the data using PCA, all groups can be discriminated from each other. As seen in Figure 2 (left), PC1 discriminates cytoplasm (Cyto) from mitochondria (Mito), while PC2 discriminates *sym1*Δ from WT. Interestingly, the shift between the two cytoplasm groups is greater than that between the mitochondria groups. To present how every single metabolite contributes to the discrimination of groups in the PCA score plot, a PCA loading plot (Figure 2, right) was applied. The individual loading values are displayed in Supplementary Table S2. Loading 1 shows how the metabolites differentiated the compartments, highlighting a group of metabolites, including several fatty acids and steroid intermediates. The metabolites in loading 2 differentiate sym1Δ from WT, among which cytidine, ornithine, citrulline, gluconic acid, orotic acid, and carbamoyl-aspartate contributed the most. To present the detailed alteration of significantly changed metabolites among groups (results of statistical analyses are displayed in Supplementary Table S3), a heat map is shown in Figure 3. The metabolites are mainly clustered into two groups, including metabolites with either higher abundance in mitochondria or cytoplasm. Subclusters can be observed in each group, presenting different regulation patterns of metabolites. According to ANOVA, lysine was the top significantly changed metabolite. Interestingly, lysine biosynthesis intermediates, 2-aminoadipate (2-AAA) and saccharopine, were accumulated. We thus focused on lysine metabolism in the following experiments.

### 3.2. Sym1Δ Cells Are Lysine Auxotroph

**Figure 4:**
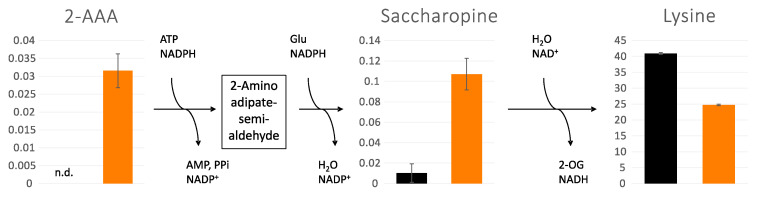
Accumulation of lysine biosynthesis intermediates, 2-aminoadipate (2-AAA) and saccharopine, and reduction of lysine in cytoplasm of WT (black) and *sym1*Δ (orange) cells. Y-Axes show normalized intensities of each metabolite in arbitrary units. n.d.: not detected, n = 3, error bars indicate standard deviation.

**Figure 5:**
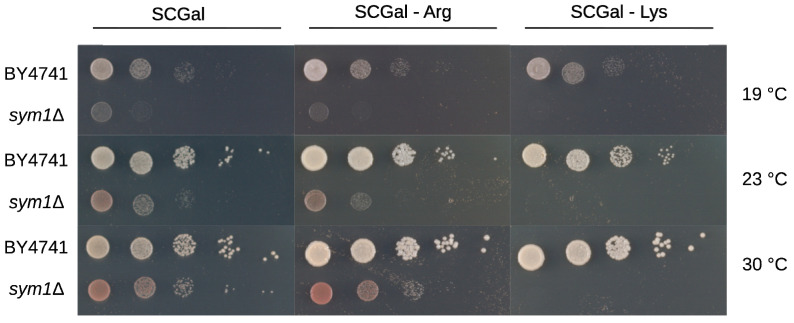
Growth test of BY4741 and *sym1*Δ on SCGal plates at 19, 23, and 30 °C for five days. BY4741 and *sym1*Δ were incubated on SCGal plate with full amino acids (**left**), without arginine (**middle**), and without lysine (**right**).

While lysine was significantly reduced in *sym1*Δ cells, the intermediates for lysine biosynthesis, 2-AAA and saccharopine, were accumulated as depicted in Figure 4. To examine the function of lysine, a growth test was investigated. Surprisingly, *sym1*Δ cells could not grow without lysine at any temperature tested (19, 23, and 30 °C), as shown in Figure 5. As a control, they could grow on both plates of synthetic medium with full amino acids (SCGal) and without arginine (SCGal-Arg). While the depletion of arginine maintained the growth with slight repression, the depletion of lysine repressed the growth completely. 

Interestingly, the fungal 2-aminoadipate pathway for lysine biosynthesis takes place in mitochondria for the first four steps and in the cytosol for the last four steps [25]. De novo biosynthesis of lysine begins with α-ketoglutarate in mitochondria. The intermediate α-ketoadipate is exported to the cytosol before the synthesis continues for four further steps to form lysine. Therefore, it is possible that any step of this synthesis pathway was inhibited due to the deletion of SYM1. However, the addition of either intermediate for lysine biosynthesis or lysine could not rescue the growth defect of *sym1*Δ cells as shown in Supplementary Figure S1.

### 3.3. Establishment of a Workflow for Detection of Unknown Metabolites

An LC–QTOF-based workflow was developed with the focus on significantly altered metabolites caused by the deletion of SYM1, supplying a strategy to reduce the data complexity. Four replicates of Mito and Cyto of WT or *sym1*Δ cells were analyzed in full scan mode. The average mass or retention time (RT) deviation was calculated based on the mass or RT deviation of all compounds. The average RT deviations of four replicates of Mito and Cyto were 0.00973 and 0.012 min, respectively, indicating a highly stable RT. The average mass deviations were 0.891 and 0.915 ppm, respectively, satisfying the requirement for the workflow. Due to different matrix effects, Mito and Cyto were processed separately.

**Figure 6:**
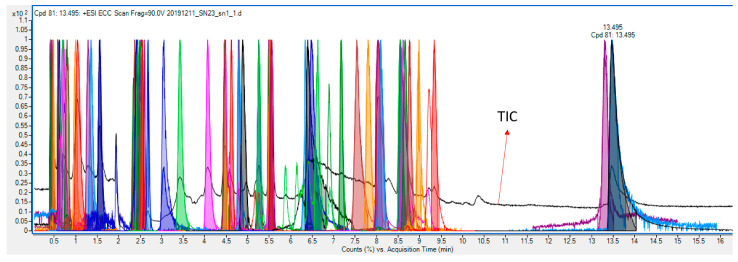
An example of a total ion chromatogram (TIC) with extracted chromatograms of compounds.

**Figure 7:**
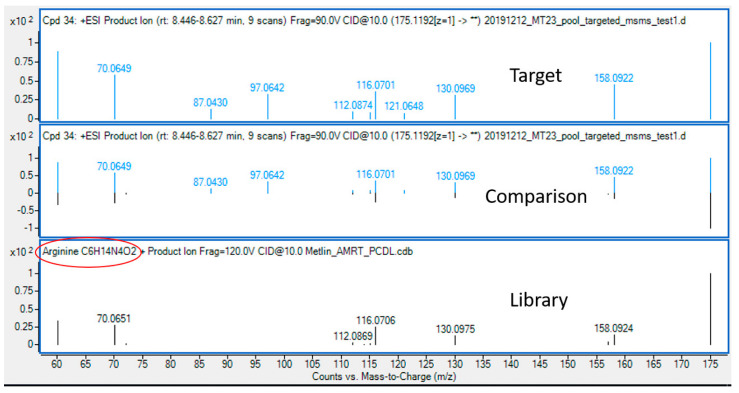
Identification of arginine through library search. The identification of metabolites was done by searching library with mass spectral information.

An example of a total ion chromatogram (TIC) with extracted chromatograms of compounds is displayed in Figure 6, showing good separation of compounds through HILIC-based chromatography. After the targeted analysis of metabolites provided in the MS-inclusion list (Supplementary Table S4), the metabolites were identified through a library search. As shown in Figure 7, the measured spectra of the target with an accurate *m*/*z* of 175.1192 and an RT of 8.03 min was compared with the spectra in the library, identifying this compound as arginine. Through library search of all the targets, 27 out of 34 and 12 out of 27 compounds were found as putative metabolites in Mito of WT and *sym1*Δ cells, respectively, whereas 47 out of 62 and 39 out of 58 compounds were found as putative metabolites in Cyto of WT and *sym1*Δ cells, respectively, as summarized in Table 1. Seven and eight metabolites could be identified through an automatic library search in Mito of WT and *sym1*Δ cells, respectively, whereas 24 and 21 could be identified in Cyto of WT and *sym1*Δ cells, respectively. However, the rest of the putative metabolites could not be identified due to the absence of spectral information, suggesting a further extension of the library.

The identified metabolites are listed in Table 2, covering amino acids and related metabolites, peptides, nucleotides, and lipids. The reduced arginine and increased citrulline, together with reduced orotic acid, correlate with the acquired data using combined methods, indicating reproducible results across different analytical techniques. Except for those metabolites that were identified already, forty-one novel metabolites could be revealed including two metabolites from the arginine metabolism pathway, 4-guanidinobutanal and 4-guanidinobutanoate; modified nucleobase and nucleoside, 1-methylguanine, 5-methyl cytosine, and 5-methylcytidine; phospholipids; modified amino acids; and di- or tri-peptides.

## 4. Discussion

Compartment-specific metabolomics provides new opportunities in research of sub-cellular metabolism. In that way, metabolic alterations in mitochondria could be observed, which could otherwise be overlooked in whole cell analyses. The isolation process of mitochondria on the other hand could potentially influence the metabolome itself. Nowadays, different isolation protocols are available which are adequate to metabolomics and for which a sustained metabolic pattern was shown [2,5]. In particular, our applied methodology had been shown to have intact membrane integrity, and metabolic patterns derived from well-characterized mutants had been conserved [2]. This method was employed to study the mitochondrial inner membrane protein Sym1 without a defined function. A combination of GC–MS profiling with targeted LC–MS has increased the coverage of metabolites. Importantly, specific metabolite distributions, for example, for fructose-6-phosphate or sedoheptulose-7-phosphate were observed matching those observed in the literature [2,5]. Our study found differentially regulated lysine metabolism and identified *sym1*Δ cells as lysine auxotroph. While the depletion of arginine maintained the growth with slight repression, the depletion of lysine repressed the growth completely. Generally, yeast cells can synthesize all twenty amino acids [26]. The knock-out of Sym1 protein makes lysine an essential amino acid, indicating that lysine biosynthesis is severely impaired. Lysine biosynthesis intermediates, 2-aminoadipate (2-AAA) and saccharopine, were accumulated, indicating an impaired lysine biosynthesis or degradation. Remarkably, accumulating saccharopine levels caused by mutations of its degrading enzyme resulted in a functional disruption of mitochondria in *Caenorhabditis elegans* [27]. Furthermore, genetically caused saccharopine accumulation in mice also resulted in mitochondrial dysfunction, especially apparent in the liver. Further, that liver dysfunction was lethal [27]. This is particularly interesting since all reported patients with MDDS caused by mutations in MPV17, the human ortholog of SYM1, presented with liver dysfunction and 91% of them with liver failure [9]. Our results might suggest a role of lysine metabolism in human MDDS, which should be addressed in future studies. Another consequence of the loss of the Sym1 protein was the reduction of pyrimidine biosynthesis intermediates, carbamoyl-aspartate and orotic acid. It is worth noticing that this strain does not contain the gene URA3, resulting in an interrupted UMP biosynthesis. Consequently, the two intermediates are not expected to be observed in both WT and the mutant. Thus, the occurrence and reduction of the two precursors may indicate that they are not only precursors for UMP but also play important roles in cellular function that need to be elucidated. 

To discover potential novel metabolites associated with this protein, a workflow was applied focusing on only significantly changed metabolites in *sym1*Δ cells in comparison to WT. This workflow is a useful strategy to reduce the data complexity while interesting targets can be discovered. Consequently, several novel metabolites could be identified including 4-guanidinobutanal and 4-guanidinobutanoate, originating from an alternative arginine degradation pathway [28]. Although there is little research done on these two metabolites in *S. cerevisiae*, their existence and reduction in the *sym1*Δ mutant indicate potential biological roles for cellular stress tolerance. The reduction of arginine and increase of citrulline in *sym1*Δ cells showed a similar effect as the heat stress-induced remodeling of arginine metabolism in WT cells [3]. This might indicate that *sym1*Δ cells were under cellular stress despite the normal growth temperature, at least at the metabolite level. Consequently, *sym1*Δ cells cannot manage to survive at 37 °C. However, it is still not clear how Sym1 is involved in cellular stress management. In future works, the compartment-specific metabolomics workflow should be applied to *sym1*Δ cells under different stress conditions, such as heat-stress, ethanol stress, or osmotic stress. Those results could then be correlated to non-metabolic stress markers, such as heat shock proteins.

Although several peptides were significantly changed in *sym1*Δ cells, no hypothesis for any biological functions can be made before the origin of the detected peptides is identified. They could be either breakdown products of proteins or free cellular peptides that may have biological functions. Except for glutamyl-cysteine, known as the precursor for glutathione, the other identified dipeptides and tripeptides do not have known biological roles. One further concern is the origin of these peptides. Although altered peptide levels have been identified in patients with liver disease [29] or non-small-cell lung cancer [30], the origin or function of these peptides was not identified. Therefore, further investigation should be made to find out the origin and the biological function of oligopeptides in cells or patients, which could help in understanding their roles in diseases and cellular mechanisms.

By applying whole cell experiment as described in [3], the existence of free peptides in cells can be examined. Therefore, further experiments are required. Nevertheless, the volume of databases is key for this workflow to identify unknown metabolites. 

Overall, compartment-specific metabolomics is a useful tool for identifying metabolic dysregulations in subcellular compartments. Still, it is worth noting that the metabolic network is far more complex to expect a single alteration as the response to the loss of a specific protein.

## Figures and Tables

**Table 1:** The number of found and identified metabolites according to the inclusion list.

	Mito			Cyto		
	Targets	Putative	Identified	Targets	Putative	Identified
WT	34	27	7	62	47	24
sym1Δ	27	12	8	58	39	21

**Table 2:** Through library search, identified metabolites that are significantly (*p*-value < 0.001) reduced or increased (FC > 3) in Mito and Cyto of *sym1*Δ cells.

Mito	
Reduced	Increased
Arginine	13E/Z-Docosenamide
Gln Lys	5-Methylcytidine
GPEtn(18:1(6Z)/18:1(6Z))[U]	5-Methylcytosine
Hypoxanthine	Asn Asp
Orotic acid	Gln Ser Met
PC(10:0/18:1(9Z))	Glutamine
Sorbitol	Frag Gln
	Ser Glu
**Cyto**	
**Reduced**	**Increased**
1-Methylguanine	5-Methylcytidine
3-Hydroxy-DL-kynurenine	5-Methylcytosine
4-Guanidinobutanal	Ala Glu
4-Guanidinobutanoate	Citrulline
4-Methylene-L-glutamine	Glu Cys
Arginine	Cys Met Tyr
Arg Gly	Glu Thr Glu
Asn Ser	Glutamine
Asn Val Ser	His Gln Gln
Gly Tyr	Ile Tyr Thr
Guanosine	Ser Gln
Methyl-myo-inositol or glucoside	Ser Glu
N-Acetyl-L-glutamate 5-semialdehyde	Ser Glu
Nα-Acetyl-L-arginine	Thr Ala Asp
PC	Val Asp Asp
PC	Isobutylglycine
Phe Ile Ala	Queuine
PS	Pyroglutamic acid
Thiamine	
Thr Asn	
Thr-Arg	
Val Gln	
Val Thr	
Val Trp	

## Data Availability

Data are displayed in the Supplementary Material.

## Supplementary Materials

The following are available online at https://www.mdpi.com/article/10.3390/cells12050692/s1, Figure S1: Phenotype test of WT and *sym1*Δ cells with addition of lysine in various concentrations (right) and intermediates of lysine biosynthesis (left). Table S1: Metabolite intensity table, Table S2: PCA loadings, Table S3: Results of statistical analysis, Table S4: Inclusion list for MSMS analysis.

## References

[1] Siess E.A., Brocks D.G., Wieland O.H. (1978). Distribution of Metabolites Between the Cytosolic and Mitochondrial Compartments of Hepatocytes Isolated from Fed Rats. Hoppe-Seyler’s Z. Physiol. Chem..

[2] Pan D., Lindau C., Lagies S., Wiedemann N., Kammerer B. (2018). Metabolic profiling of isolated mitochondria and cytoplasm reveals compartment-specific metabolic responses. Metabolomics.

[3] Pan D., Wiedemann N., Kammerer B. (2019). Heat Stress-Induced Metabolic Remodeling in Saccharomyces cerevisiae. Metabolites.

[4] Roede J.R., Park Y., Li S., Strobel F.H., Jones D.P. (2012). Detailed Mitochondrial Phenotyping by High Resolution Metabolomics. PLoS ONE.

[5] Chen W.W., Freinkman E., Wang T., Birsoy K., Sabatini D.M. (2016). Absolute Quantification of Matrix Metabolites Reveals the Dynamics of Mitochondrial Metabolism. Cell.

[6] Matuszczyk J.-C., Teleki A., Pfizenmaier J., Takors R. (2015). Compartment-specific metabolomics for CHO reveals that ATP pools in mitochondria are much lower than in cytosol. Biotechnol. J..

[7] Spinazzola A., Viscomi C., Fernandez-Vizarra E., Carrara F., D’Adamo A.P., Calvo S., Marsano R.M., Donnini C., Weiher H., Strisciuglio P. (2006). MPV17 encodes an inner mitochondrial membrane protein and is mutated in infantile hepatic mitochondrial DNA depletion. Nat. Genet..

[8] Suomalainen A., Isohanni P. (2010). Mitochondrial DNA depletion syndromes—Many genes, common mechanisms. Neuromuscul. Disord..

[9] El-Hattab A.W., Wang J., Dai H., Almannai M., Staufner C., Alfadhel M., Gambello M.J., Prasun P., Raza S., Lyons H.J. (2018). *MPV17*-related mitochondrial DNA maintenance defect: New cases and review of clinical, biochemical, and molecular aspects. Hum. Mutat..

[10] Löllgen S., Weiher H. (2015). The role of the Mpv17 protein mutations of which cause mitochondrial DNA depletion syndrome (MDDS): Lessons from homologs in different species. Biol. Chem..

[11] Dallabona C., Marsano R.M., Arzuffi P., Ghezzi D., Mancini P., Zeviani M., Ferrero I., Donnini C. (2010). Sym1, the yeast ortholog of the MPV17 human disease protein, is a stress-induced bioenergetic and morphogenetic mitochondrial modulator. Hum. Mol. Genet..

[12] Antonenkov V.D., Isomursu A., Mennerich D., Vapola M.H., Weiher H., Kietzmann T., Hiltunen J.K. (2015). The Human Mitochondrial DNA Depletion Syndrome Gene MPV17 Encodes a Non-selective Channel That Modulates Membrane Potential. J. Biol. Chem..

[13] Krauss J., Astrinides P., Frohnhöfer H.G., Walderich B., Nüsslein-Volhard C. (2013). Transparent, a gene affecting stripe formation in Zebrafish, encodes the mitochondrial protein Mpv17 that is required for iridophore survival. Biol. Open.

[14] Trott A., Morano K.A. (2004). *SYM1* Is the Stress-Induced *Saccharomyces cerevisiae* Ortholog of the Mammalian Kidney Disease Gene *Mpv17* and Is Required for Ethanol Metabolism and Tolerance during Heat Shock. Eukaryot. Cell.

[15] Weiher H., Noda T., Gray D.A., Sharpe A.H., Jaenisch R. (1990). Transgenic mouse model of kidney disease: Insertional inactivation of ubiquitously expressed gene leads to nephrotic syndrome. Cell.

[16] Rosa I.D., Cámara Y., Durigon R., Moss C.F., Vidoni S., Akman G., Hunt L., Johnson M.A., Grocott S., Wang L. (2016). MPV17 Loss Causes Deoxynucleotide Insufficiency and Slow DNA Replication in Mitochondria. PLoS Genet..

[17] Alonzo J.R., Venkataraman C., Field M., Stover P.J. (2018). The mitochondrial inner membrane protein MPV17 prevents uracil accumulation in mitochondrial DNA. J. Biol. Chem..

[18] Gilberti M., Baruffini E., Donnini C., Dallabona C. (2018). Pathological alleles of MPV17 modeled in the yeast Saccharomyces cerevisiae orthologous gene SYM1 reveal their inability to take part in a high molecular weight complex. PLoS ONE.

[19] Ammar S., Abidi J., Luca S.V., Boumendjel M., Skalicka-Woźniak K., Bouaziz M. (2020). Untargeted metabolite profiling and phytochemical analysis based on RP-HPLC-DAD-QTOF-MS and MS/MS for discovering new bioactive compounds in *Rumex algeriensis* flowers and stems. Phytochem. Anal..

[20] Rocchetti G., Gallo A., Nocetti M., Lucini L., Masoero F. (2020). Milk metabolomics based on ultra-high-performance liquid chromatography coupled with quadrupole time-of-flight mass spectrometry to discriminate different cows feeding regimens. Food Res. Int..

[21] Armitage E.G., Barbas C. (2014). Metabolomics in cancer biomarker discovery: Current trends and future perspectives. J. Pharm. Biomed. Anal..

[22] Lagies S., Schlimpert M., Braun L.M., Kather M., Plagge J., Erbes T., Wittel U.A., Kammerer B. (2019). Unraveling altered RNA metabolism in pancreatic cancer cells by liquid-chromatography coupling to ion mobility mass spectrometry. Anal. Bioanal. Chem..

[23] De Vos R.C.H., Moco S., Lommen A., Keurentjes J., Bino R.J., Hall R. (2007). Untargeted large-scale plant metabolomics using liquid chromatography coupled to mass spectrometry. Nat. Protoc..

[24] Pang Z., Chong J., Zhou G., de Lima Morais D.A., Chang L., Barrette M., Gauthier C., Jacques P.-É., Li S., Xia J. (2021). MetaboAnalyst 5.0: Narrowing the gap between raw spectra and functional insights. Nucleic Acids Res..

[25] Zabriskie T.M., Jackson M.D. (2000). Lysine biosynthesis and metabolism in fungi. Nat. Prod. Rep..

[26] Strathern J.N., Jones E.W., Broach J.R. (1981–1982). The Molecular Biology of the Yeast Saccharomyces.

[27] Zhou J., Wang X., Wang M., Chang Y., Zhang F., Ban Z., Tang R., Gan Q., Wu S., Guo Y. (2019). The lysine catabolite saccharopine impairs development by disrupting mitochondrial homeostasis. J. Cell Biol..

[28] Romagnoli G., Verhoeven M.D., Mans R., Rey Y.F., Bel-Rhlid R., Broek M.D., Seifar R.M., Pierick A.T., Thompson M., Müller V. (2014). An alternative, arginase-independent pathway for arginine metabolism in *Kluyveromyces lactis* involves guanidinobutyrase as a key enzyme. Mol. Microbiol..

[29] Soga T., Sugimoto M., Honma M., Mori M., Igarashi K., Kashikura K., Ikeda S., Hirayama A., Yamamoto T., Yoshida H. (2011). Serum metabolomics reveals γ-glutamyl dipeptides as biomarkers for discrimination among different forms of liver disease. J. Hepatol..

[30] Wu M., Xu Y., Fitch W.L., Zheng M., Merritt R.E., Shrager J.B., Zhang W., Dill D.L., Peltz G., Hoang C.D. (2013). Liquid chromatography/mass spectrometry methods for measuring dipeptide abundance in non-small-cell lung cancer. Rapid Commun. Mass Spectrom..

