# Supplementary material for: Mitochondrial Metabolomics of Sym1-Depleted Yeast Cells Revealed Them to Be Lysine Auxotroph
Source: Cells. 2023 Feb 22;12(5):692. doi: 10.3390/cells12050692 (PMC10000845; doi:10.3390/cells12050692)
Supplement: Supplementary file 1 [file cells-12-00692-s001.zip › Supplementary Figure S1.pdf]

cells

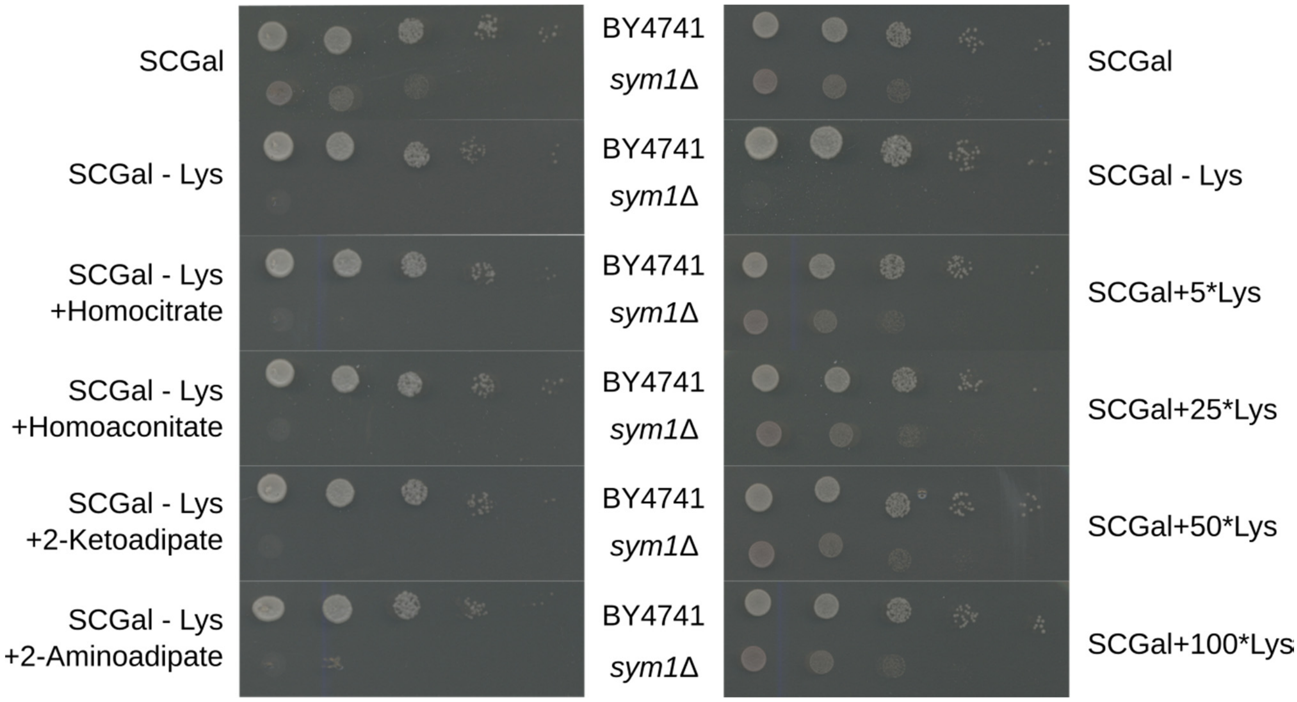

Figure S1: Phenotype test of WT and *sym1*Δ cells with addition of lysine in various concentrations (right) and intermediates of lysine biosynthesis (left).
